# Elevated malondialdehyde levels in sepsis - something to 'stress' about?

**DOI:** 10.1186/cc13786

**Published:** 2014-03-19

**Authors:** Scott L Weiss, Clifford S Deutschman

**Affiliations:** 1 Division of Critical Care Medicine, Department of Anesthesia and Critical Care, The Children’s Hospital of Philadelphia, University of Pennsylvania Perelman School of Medicine, Philadelphia, PA 19104, USA; 2 Department of Anesthesiology and Critical Care, University of Pennsylvania Perelman School of Medicine, Philadelphia, PA 19104, USA

## Abstract

Oxidative stress has been postulated as a mechanism of organ dysfunction - and thus a potential therapeutic target - in sepsis. Lorente and colleagues report increased serum levels of malondialdehyde, a biomarker of oxidative stress-induced lipid peroxidation, in adults with severe sepsis, particularly in non-survivors. While survivors exhibited a decrease in serum malondialdehyde over time, the elevation was sustained in non-survivors. These findings suggest that there is increased oxidative stress in sepsis and that membrane lipids in particular are targeted by free radical species. Further study is required to validate the utility of malondialdehyde as a prognostic biomarker in sepsis and to determine a role for antioxidant therapy.

## 

Oxidative stress has been implicated in the pathogenesis of sepsis-induced organ dysfunction and thus represents a potential therapeutic target. However, because reactive oxygen and nitrogen species have very short half-lives, demonstration of free radical formation in human samples is exceedingly difficult. Therefore, measurements of oxidative stress have relied on quantifying byproducts of macromolecule oxidation. In a previous issue of *Critical Care*, Lorente and colleagues demonstrated increased levels of malondialdehyde (MDA), a marker of lipid peroxidation, in the serum of adults with severe sepsis [1]. Non-survivors exhibited higher MDA levels than non-survivors. MDA levels were increased irrespective of site of infection, type of micro-organism, or dysfunctional organ system, suggesting that lipid peroxidation was attributable to the general host response rather than a pathogen- or organ-specific reaction. In survivors, serum MDA normalized within 4 days while levels remained elevated in non-survivors. Increased MDA was independently associated with 30-day and 6-month all-cause mortality. What can we hope to learn from this study and how can we use these data to improve outcomes for patients with sepsis?

The presence of high serum MDA levels supports the hypothesis that increased oxidative stress, particularly lipid peroxidation, contributes to sepsis pathophysiology. Oxidative stress occurs when free radical production exceeds the antioxidant capacity of endogenous molecules such as glutathione, superoxide dismutase, thioreduxin and vitamin E. Lipid peroxidation results when fatty acids come into contact with reactive oxygen species (ROS), producing a series of reactive aldehydes, including MDA. Mitochondria are a major source of intracellular ROS because incomplete reduction of oxygen to water through the electron transport chain produces superoxide and hydrogen peroxide [2]. Normally, an estimated 1% of oxygen consumption is directed to ROS. These molecules play an essential role in cell signaling and immune defense [3]. In sepsis, however, uncoupled oxidative phosphorylation and depleted antioxidant stores are postulated mechanisms leading to an enhanced ROS burden [4,5]. The combination of increased superoxide and up-regulated nitric oxide production in sepsis leads to enhanced production of peroxynitrite and other toxic reactive nitrogen species (RNS) [2]. The net result is accumulated damage to lipids, proteins, and nucleic acids that may impede cellular function. In particular, oxidative injury to lipids within plasma and mitochondrial membranes can alter permeability and impair membrane-bound receptors and enzymes. MDA and other reactive aldehydes are among the toxic species that can disrupt protein structure and function [6]. Because aldehydes are released into the blood when cells are damaged by lipid peroxidation, serum MDA has been touted as an indirect marker of oxidative stress [7].

Lorente and colleagues hypothesized that serum MDA, as a biomarker of oxidative stress, could provide prognostic information about mortality risk in sepsis. While MDA levels were elevated in non-survivors, the predictive utility of this biomarker in their study was disappointing. The area under the receiver operating characteristic curve for serum MDA to predict 30-day mortality was a modest 0.67 (95% CI 0.61 to 0.72). The MDA cutpoint suggested by the authors of 4.11 nmol/ml exhibited poor sensitivity (42%, 95% CI 32 to 51%) and only moderate specificity (82%, 95% CI 76 to 87%) for clinical use. Even with 34% overall mortality in their study, about half of those in whom MDA predicted death (that is, MDA ≥4.11 nmol/ml) ultimately survived. The considerable overlap between sepsis survivors and non-survivors limits the utility of serum MDA levels in clinical practice, as is the case with many other purported sepsis biomarkers [8].

Several explanations may account for the unreliability of serum MDA levels in sepsis. First, lipid peroxidation is not specific to sepsis and may be affected by comorbid conditions, diet, and lifestyle behaviors. Increased blood MDA levels occur in smokers [9] and patients with diabetes mellitus and hypertriglyceridemia [10,11]. Second, the technique used by the authors to measure MDA has important limitations. Although the thiobarbituric acid-reactive substances (TBARS) assay is accepted as an index of oxidative stress, this method does not specifically measure MDA or lipid peroxidation and the presence of other aldehydes may confound the results [7]. In addition, not all TBARS, including MDA, are derived from lipid peroxidation. Use of high-performance liquid chromatography to isolate MDA would improve specificity beyond the spectrophotometric measurements used in this study [12].

Perhaps combining measurements of MDA with other oxidative stress biomarkers and additional clinical variables could improve outcome prediction for septic patients. However, whether oxidative stress causes or propagates the pathogenesis of sepsis remains unclear [13]. Although verifying the occurrence of oxidative stress is an important step, this observation cannot determine whether lipid peroxidation or other ROS/RNS-related damage is an important mechanism of sepsis-induced organ injury. For decades, the observed pro-inflammatory response characteristic of early sepsis was believed to be the primary cause of organ injury but, to date, trials of anti-inflammatory agents have been consistently negative [14]. Similarly, use of antioxidant therapies in sepsis remains an attractive but unproven strategy. While antioxidant supplementation with vitamin E, selenium, N-acetylcysteine, and other compounds can reduce oxidative stress, clinical trials have failed to convincingly demonstrate an outcome benefit [15]. Anti-oxidants targeted to the mitochondria, a primary site of ROS/RNS production, have shown promise in preclinical studies and may be better suited for use in critically ill patients [4,5]. Future studies should consider if, as Lorente and colleagues suggest, biomarkers can identify patients with increased oxidative stress who may derive the greatest benefit from anti-oxidant therapy in sepsis.

## Abbreviations

MDA: Malondialdehyde; ROS: Reactive oxygen species; RNS: Reactive nitrogen species; TBARS: Thiobarbituric acid-reactive substances.

## Competing interests

The authors declare that they have no competing interests.
